# Supplementary material for: Molecular Epidemiology and Baseline Resistance of Hepatitis C Virus to Direct Acting Antivirals in Croatia
Source: Pathogens. 2022 Jul 19;11(7):808. doi: 10.3390/pathogens11070808 (PMC9323280; doi:10.3390/pathogens11070808)
Supplement: Supplementary file 1 [file pathogens-11-00808-s001.zip › pathogens-1807483-supplementary.pdf]

Table S1. Outer primers for NS3, NS5A and NS5B amplification.

REGION	SUBTYPE	PRIMER DIRECTION	NUCLEOTIDE SEQUENCE (5'–3')	GENOME POSITION*	REFERENCES
NS3	1a	forward	ATGGAGACCAAGCTCATCACGTGGG	3276–3300	Dietz et al., 2015
		reverse	ACCCGCCGTCGGCAAGGAACTTGCCGTA	4227–4254	Dietz et al., 2015
	1b	forward	TGGAGACYAAGMTCATYACSTGGG	3277–3300	Gozlan et al., 2017
		reverse	CCARGACYGTGCCRATGCCCA	4321–4339	Zhou et al., 2017
	3a	forward	TACGACCACCTAGCGCCAATG	3276–3297	primer design (Ye et al., 2012)
		reverse	GGGACCTTTGTGCTCTTAC	4045–4063	primer design (Ye et al., 2012)
NS5A	1a	forward	ACATCCTTGCAGGGTATGG	5890–5908	Dietz et al., 2018
		reverse	GACCACAGGTGGTTCGTAG	7256–7274	Dietz et al., 2018
	1b	forward	TGGATGAACCGGCTGATAG	6084–6102	McCormick et al., 2015
		reverse	CCACAGGAGRTTGGCCTC	7023–7040	McCormick et al., 2015
	3a	forward	TGGATGAACAGGCTCATYGC	6084–6103	McCormick et al., 2015
		reverse	ATGTTRCTGCCCATCTCTTG	7044–7063	McCormick et al., 2015
NS5B	1a	forward	CTYAGCGACGGRTCRT	7539–7554	Peres-da-Silva, de Almeida and Lampe, 2017
		reverse	TCACGGGTRAGGTARTAGAC	8742–8761	Peres-da-Silva, de Almeida and Lampe, 2017
	1b	forward	TCYTGGTCTACYGTRAG	7551–7568	Peres-da-Silva, de Almeida and Lampe, 2017
		reverse	AGGARCATGATGTTATCARCTC	8682–8703	Peres-da-Silva, de Almeida and Lampe, 2017
	3a	forward	GCTCGTCTATGCCTCCTCTC	7497–7517	primer design (Ye et al., 2012)
		reverse	AAGGTCGTAAGTGGCCTGTG	8673–8693	primer design (Ye et al., 2012)
	1a/1b/3a	forward	TATGAYACCCGCTGYTTYGA	8256–8275	Akaberi et al., 2018
		reverse	GGGCAYGHGACABGCTGTGA	9284–9303	Akaberi et al., 2018

*: according to H77 reference sequence (*GenBank* accession number: AF009606)

Table S2. Inner primers for nested NS3, NS5A and NS5B amplification and seqeuncing.

REGION	SUBTYPE	PRIMER DIRECTION	NUCLEOTIDE SEQUENCE (5'-3')	GENOME POSITION*	REFERENCES
NS3	1a	forward	CGAYGGAATGGTCTCCAAG	3386–3404	Nejabat et al., 2019
		reverse	CRGCAACRGAGGGGTTGAG	4101–4119	Nejabat et al., 2019
	1b	forward	ACSGCRGCRTGYGGGGAC	3309–3326	Vallet et al., 2011
		reverse	GTGCTCTTRCCGCTRCCRGT	4035–4054	Vallet et al., 2011
	3a	forward	ATACAGCGGCTTGCGGAG	3307–3325	primer design (Ye et al., 2012)
		reverse	GCAGGAGGAGTTGAATTGTC	3975–3995	primer design (Ye et al., 2012)
NS5A	1a	forward	TGGATGAACCGGCTGATAG	6084–6102	Dietz et al., 2018
		reverse	ATGTTGCCGCCCATCTC	7047–7063	Dietz et al., 2018
	1b	forward	TCCCCCACGCACTAYGTG	6129–6146	McCormick et al., 2015
		reverse	CTRGCYGARGAGCTGGCC	6935–6952	McCormick et al., 2015
	3a	forward	GCTCATCGCGTTCGCATCC	6095–6114	primer design (Ye et al., 2012)
		reverse	CCTATGCGTCTGGCAAGTGG	7005–7025	primer design (Ye et al., 2012)
NS5B	1a	forward	TCGTGTGYTGCTCRATG	7591–7607	Peres-da-Silva, de Almeida and Lampe, 2017
		reverse	TACCTGGTCATAGCCTCC	8621–8638	Peres-da-Silva, de Almeida and Lampe, 2017
	1b	forward	TCYTGGTCTACYGTRAG	7551–7568	Peres-da-Silva, de Almeida and Lampe, 2017
		reverse	CCTAGTCATAGCCTCCGT	8619–8636	Peres-da-Silva, de Almeida and Lampe, 2017
	3a	forward	CGACTCTTGGTCCACCGTTAG	7590–7611	primer design (Ye et al., 2012)
		reverse	GCTCTCAGGGCTGCTCTATC	8595–8615	primer design (Ye et al., 2012)
	1a/1b/3a	forward	ACCCGCTGYTTYGACTCVAC	8262–8281	Akaberi, 2018
		reverse	GACASGCTGWGATADATGTC	9276–9295	Akaberi, 2018

*: according to H77 reference sequence (*GenBank* accession number: AF009606

Table S3. Representative HCV reference sequences used for phylogenetic analyses.

Genotype	Subtype	GenBank Accession Number
1	1a	AF009606
		M62321
		M67463
		HQ850279
		EF407457
	1b	D90208
		M58335
		EU781827
		EU781828
	1c	D14853
		AY051292
		AY651061
	1d	KJ439768
	1e	KC248194
	1g	AM910652
	1h	KC248198
		KC248199
	1i	KJ439772
	1j	KJ439773
	1k	KJ439774
	1l	KC248193
		KC248197
		KC248196
	1m	KJ439778
		KJ439782
	1n	KJ439781
		KJ439775
	1o	KJ439779
		MH885469
2	2a	D00944
		AB047639
		HQ639944
	2b	D10988
		AB030907
	2c	D50409
		JX227949
	2d	JF735114
	2a	JF735120
	2f	KC844042
		KC844050
	2j	DQ155561
		HM777358
		JF735113
		HM777359
	2k	AB031663
		JX227953
	2m	JF735111

		JX227967
	2k	FN666428
		FN666429
	2r	JF735115
	2t	KC197238
	2u	JF735112
3	3a	D17763
		D28917
		X76918
		JN714194
	3b	D49374
		JQ065709
	3d	KJ470619
	3e	KJ470618
	3d	JX227954
		JF735123
	3h	JF735126
		JF735121
	3i	FJ407092
		JX227955
	3k	D63821
		JF735122
4	4a	Y11604
		DQ988074
		DQ418789
	4b	FJ462435
	4c	FJ462436
	4d	DQ418786
		FJ462437
		EU392172
	4f	EF589161
		EU392175
		EU392174
	4g	FJ462432
		JX227971
	4k	EU392173
		FJ462438
		EU392171
	4l	FJ839870
		JX227957
	4m	FJ462433
		JX227972
	4n	FJ462441
		JX227970
	4o	FJ462440
		JX227977
	4p	FJ462431
	4q	FJ462434
	4r	FJ462439
		JX227976

	4s	JF735136
	4t	FJ839869
	4v	HQ537009
		JX227959
		HQ537008
		JX227960
	4w	FJ025855
		FJ025856
5	5a	AF064490
		Y13184
6	6a	Y12083
		AY859526
		HQ639936
		EU246930
	6b	D84262
	6c	EF424629
	6d	D84263
	6e	DQ314805
		EU246932
	6f	DQ835760
		EU246936
	6g	D63822
		DQ314806
	6h	D84265
	6i	DQ835770
		DQ835762
	6j	DQ835769
		DQ835761
	6k	D84264
	6l	EF424628
		JX183556
	6m	DQ835767
		DQ835766
	6n	DQ278894
		DQ835768
		EU246938
	6o	EF424627
		EU246934
	6p	EF424626
	6q	EF424625
	6r	EU408328
	6s	EU408329
	6t	EF632071
	6t	EU246939
	6u	EU246940
	6v	EU798760
		EU798761
	6w	DQ278892
		EU643834
		EU643836

7	7a	EF108306
	7b	KX092342
8	8a	MH590698
		MH590699
		MH590700
		MH590701

Table S4. Log (BF) calculations of various combinations of three demographic models of population growth and three models of molecular clock for subtype 1a, 1b and 3a sequences.

subtype 1a								
log (BF)	SMC + CP	SMC + EP	SMC + BS	RMC/LD + CP	RMC/LD + EP	RMC/LD + BS	RMC/ED + EP	RMC/ED + BS
SMC + CP	0	-86.8	-108.8	-111.9	-196.7	-208.2	-148.3	-160.2
SMC + EP	86.8	0	-22	-25.1	-109.9	-121.4	-61.6	-73.5
SMC + BS	108.8	22	0	-3.1	-87.9	-99.4	-39.6	-51.5
RMC/LD + CP	111.9	25.1	3.1	0	-84.6	-96.3	-132.8	-48.4
RMC/LD + EP	196.7	109.9	87.9	84.6	0	-11.5	48.3	36.4
RMC/LD + BS	208.2	121.4	99.4	96.3	11.5	0	59.8	47.9
RMC/ED + EP	148.3	61.6	39.6	132.8	-48.3	-59.8	0	-11.9
RMC/ED + BS	160.2	73.5	51.5	48.4	-36.4	-47.9	11.9	0
**subtype 1b**								
log (BF)	SMC + CP	SMC + EP	SMC + BS	RMC/LD + CP	RMC/LD + EP	RMC/LD + BS	RMC/ED + EP	RMC/ED + BS
SMC + CP	0	-80	-101.1	-49.3	-134.1	-157.3	-89.8	-98.1
SMC + EP	80	0	-21.1	30.7	-54.2	-77.4	-9.8	-18.1
SMC + BS	101.1	21.1	0	51.8	-33	-56.2	11.3	3.1
RMC/LD + CP	49.3	-30.7	-51.8	0	-84.8	-108	-41.5	-48.7
RMC/LD + EP	134.1	54.2	33.1	84.8	0	-23.2	44.4	36.1
RMC/LD + BS	157.3	77.4	56.2	108	23.2	0	67.6	59.3
RMC/ED + EP	89.8	9.8	-11.3	41.5	-44.4	-67.6	0	-8.3
RMC/ED + BS	98.1	18.1	-3.1	48.7	-36.1	-59.3	8.3	0
**subtype 3a**								
log (BF)	SMC + CP	SMC + EP	SMC + BS	RMC/LD + CP	RMC/LD + EP	RMC/LD + BS	RMC/ED + EP	RMC/ED + BS
SMC + CP	0	-105.3	-121.1	-79.4	-185.9	-214.2	-142.1	***
SMC + EP	105.3	0	-15.8	25.9	-80.7	-109	-36.8	***
SMC + BS	121.1	15.8	0	41.7	-64.8	-93.1	-21	***
RMC/LD + CP	79.4	-25.8	-41.7	0	-106.5	-134.8	-62.7	***
RMC/LD + EP	185.9	80.7	64.8	106.5	0	-28.3	43.9	***
RMC/LD + BS	214.2	109	93.1	134.8	28.3	0	72.2	***
RMC/ED + EP	142.1	36.8	21	62.7	-43.9	-72.2	0	***
RMC/ED + BS	***	***	***	***	***	***	***	***

BF: Bayes factor; SMC: strict molecular clock; RMC/LD: relaxed molecular clock with an uncorrelated log normal rate distribution; RMC/ED: relaxed molecular clock with an uncorrelated exponential rate distribution; CP: constant population; EP:exponential population; BS: Bayesian skyline; ***: model did not converge

a)

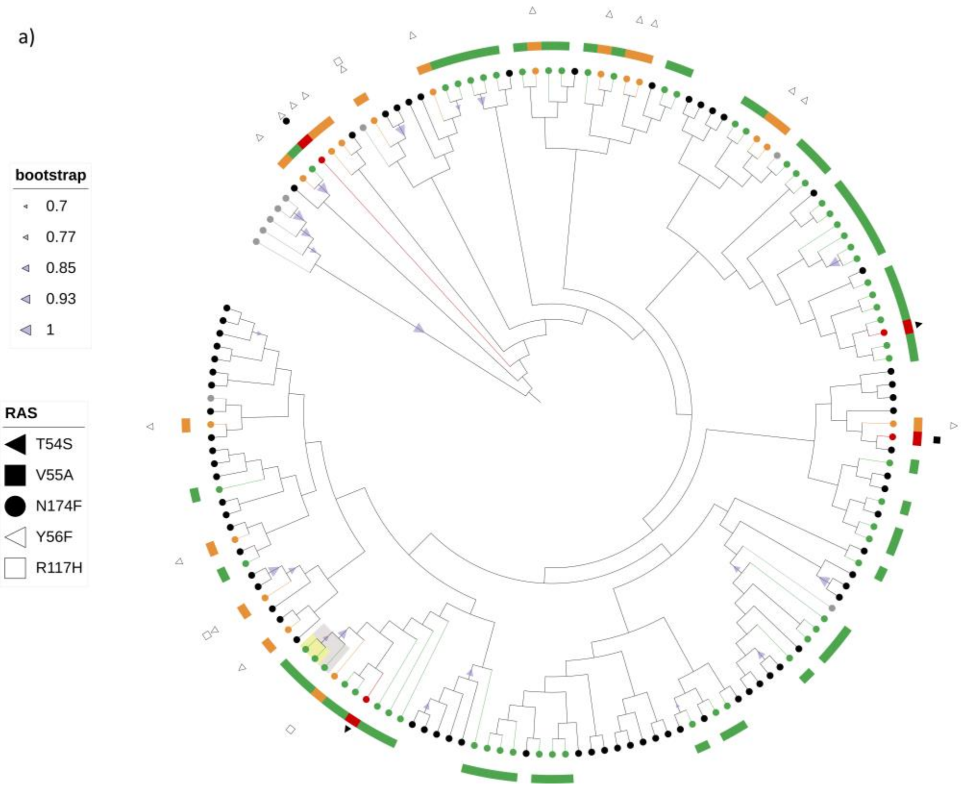

b)

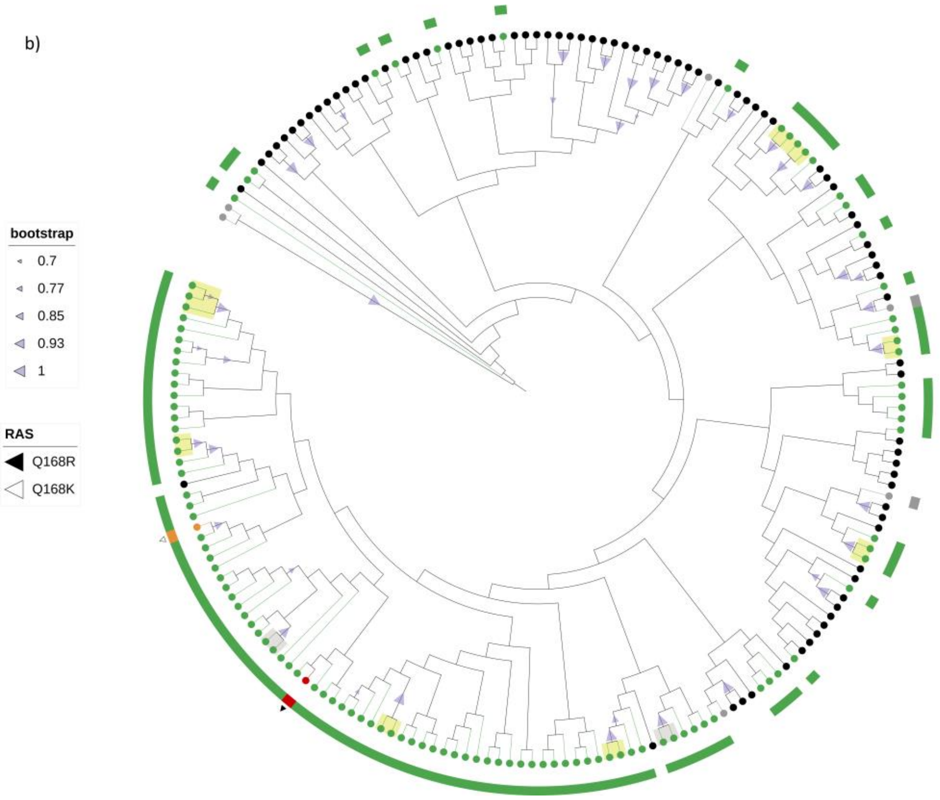

Figure S1. Maximum likelihood phylogenetic analysis of the NS3 gene sequences of a) HCV 1b subtype constructed with GTR+G+I model b) HCV 3a subtype constructed with K2+G+I model. Bootstrap values between 70 and 100% are displayed at the branch nodes as blue triangles with size corresponding to magnitude of bootstrap. Branches of two most similar control sequences per each local sequence obtained by searching the BLAST database and removing duplicates are colored black. Branches of Croatian sequences without RAS are colored green, sequences with RAS conferring resistance to DAA are colored red, and sequences with RAS associated with reduced susceptibility to DAA

are colored orange. Reference sequences are colored gray. All identified RAS are positioned on the phylogenetic tree along with the corresponding sequences. Resistance conferring RAS are marked with filled symbols, while RAS causing reduced susceptibility to at least one DAA are marked with open symbols. Identified transmission pairs are highlighted grey, while transmission pairs identified consistently across all genomic regions are highlighted yellow.

a)

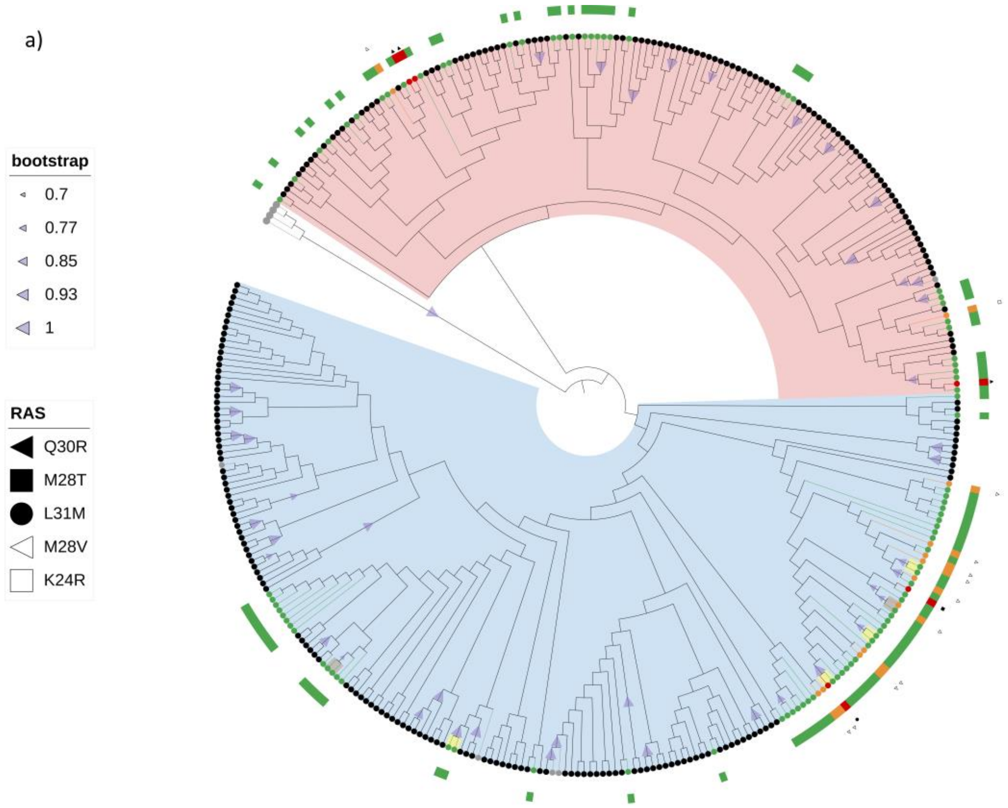

b)

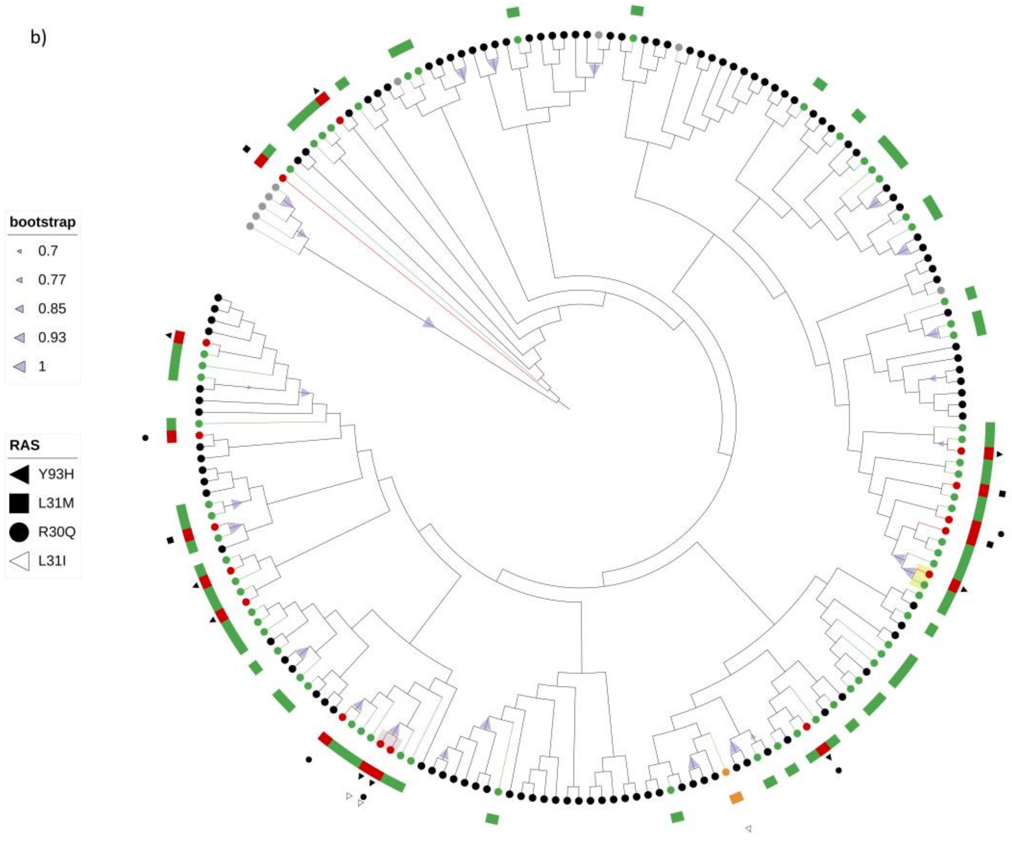

c)

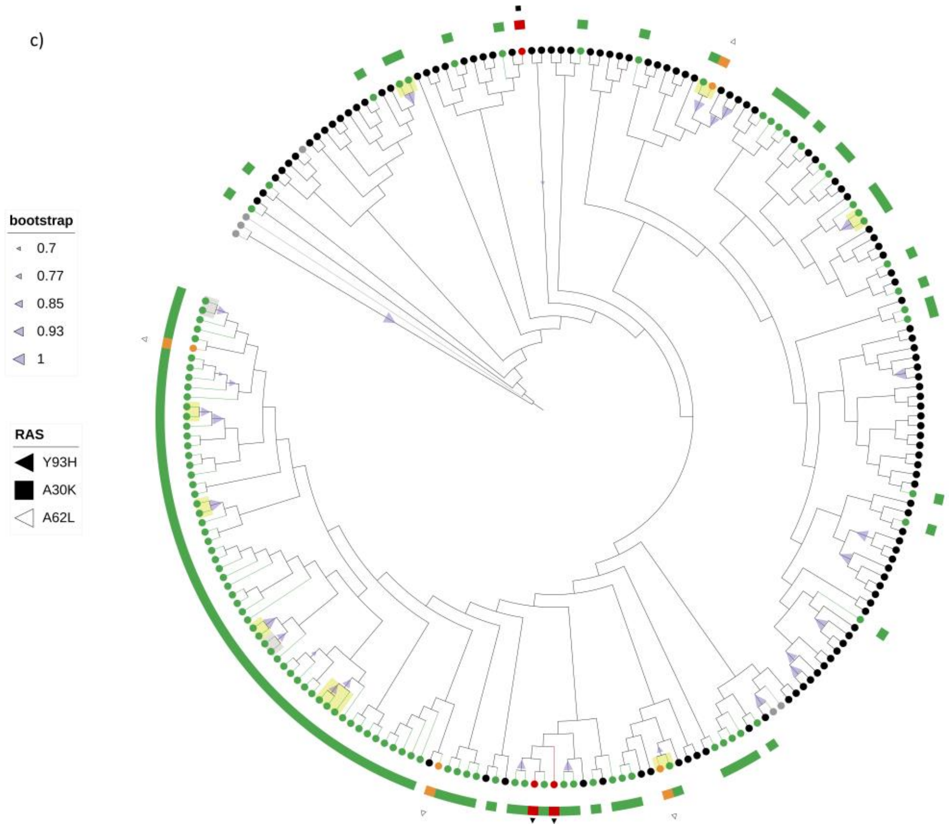

Figure S2. Maximum likelihood phylogenetic analysis of the NS5A gene sequences of a) HCV 1a subtype constructed with T92+G+I model b) HCV 1b subtype constructed with T92+G+I model c) HCV 3a subtype constructed with K2+G+I model. Bootstrap values between 70 and 100% are displayed at the branch nodes as blue triangles with size corresponding to magnitude of bootstrap. Branches of two most similar control sequences per each local sequence obtained by searching the BLAST database and removing duplicates are colored black. Branches of Croatian sequences without RAS are colored green, sequences with RAS conferring resistance to DAA are colored red, and sequences with RAS associated with reduced susceptibility to DAA are colored orange. Reference sequences are colored gray. Clade I sequences are highlighted blue, while clade II sequences are highlighted pink. All identified RAS are positioned on the phylogenetic tree along with the corresponding sequences. Resistance conferring RAS are marked with filled symbols, while RAS causing reduced susceptibility to at least one DAA are marked with open symbols. Identified transmission pairs are highlighted grey, while transmission pairs identified consistently across all genomic regions are highlighted yellow.

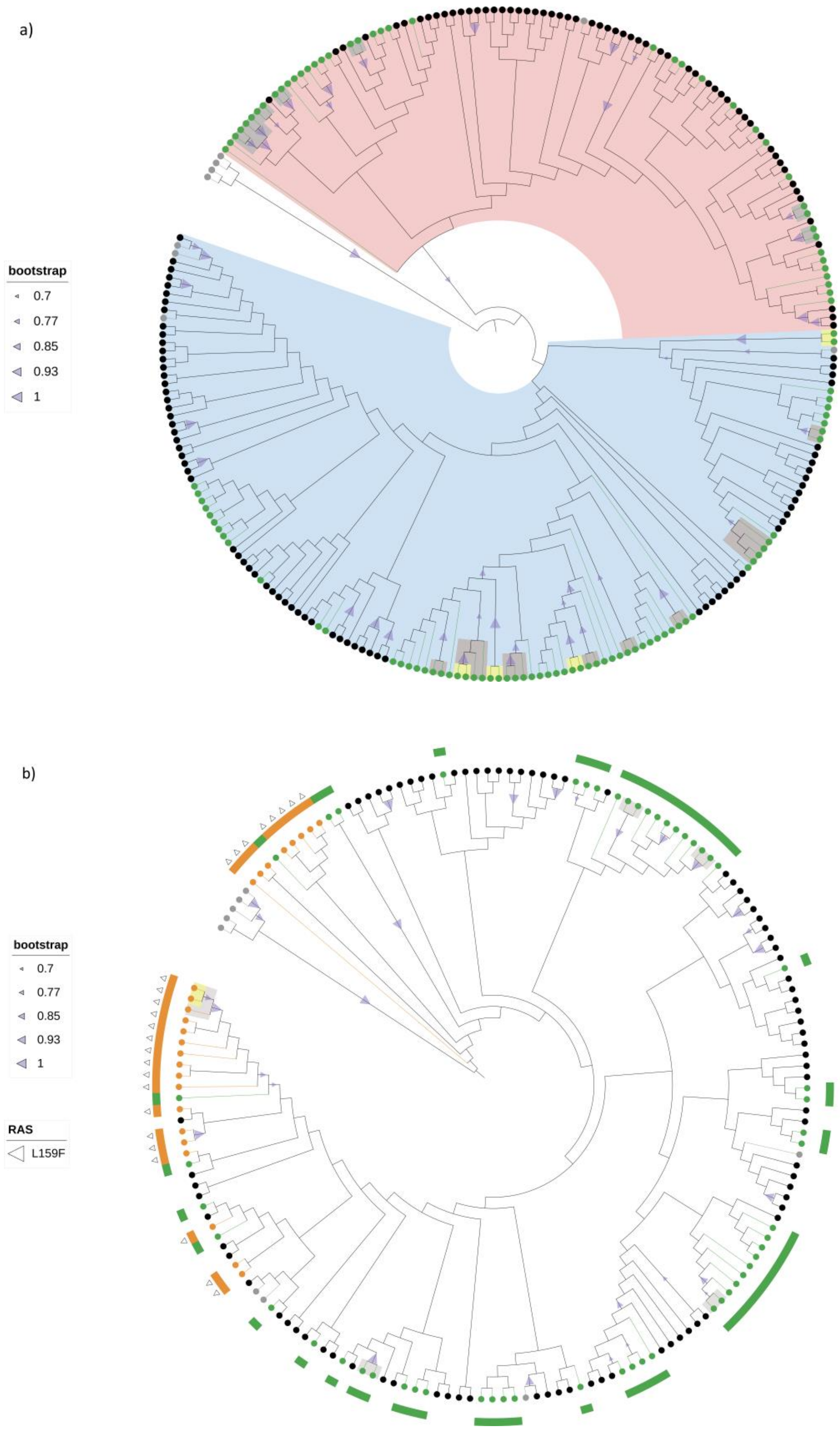

a)
bootstrap
0.7
0.77
0.85
0.93
1
b)
bootstrap
0.7
0.77
0.85
0.93
1
RAS
L159F

c)

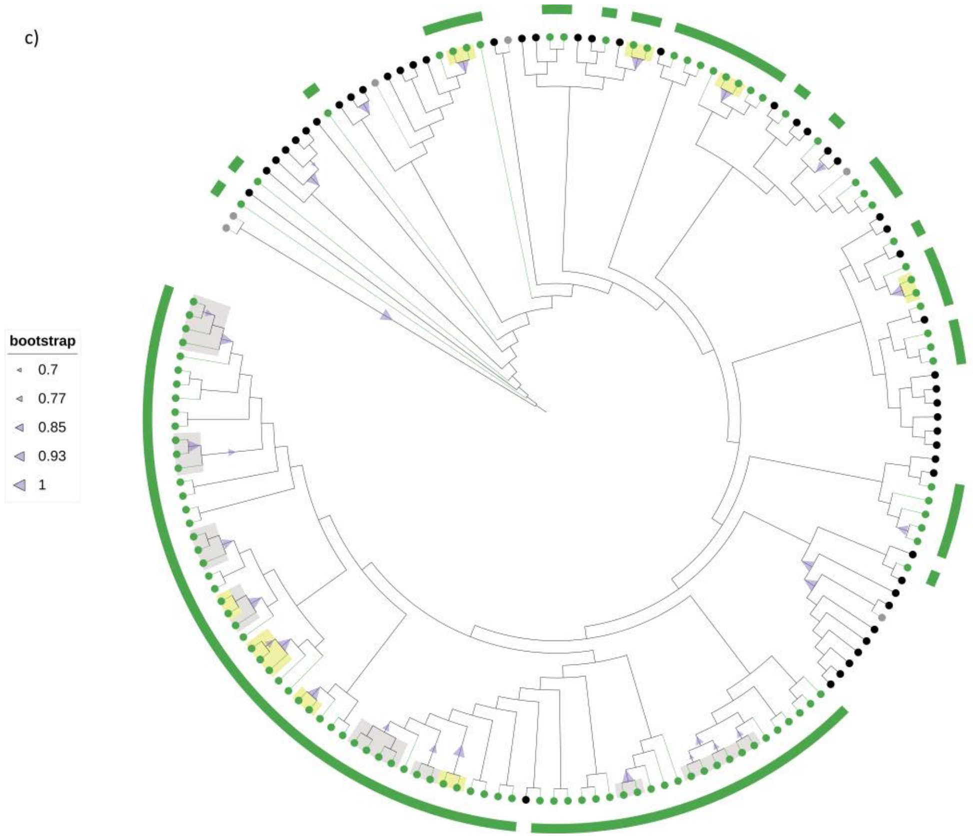

Figure S3. Maximum likelihood phylogenetic analysis of the NS5B gene sequences of a) HCV 1a subtype constructed with GTR+G+I model b) HCV 1b subtype constructed with K2+G+I model c) HCV 3a subtype constructed with K2+G+I model. Bootstrap values between 70 and 100% are displayed at the branch nodes as blue triangles with size corresponding to magnitude of bootstrap. Branches of two most similar control sequences per each local sequence obtained by searching the BLAST database and removing duplicates are colored black. Branches of Croatian sequences without RAS are colored green, sequences with RAS conferring resistance to DAA are colored red, and sequences with RAS associated with reduced susceptibility to DAA are colored orange. Reference sequences are colored gray. Clade I sequences are highlighted blue, while clade II sequences are highlighted pink. All identified RAS are positioned on the phylogenetic tree along with the corresponding sequences. Resistance conferring RAS are marked with filled symbols, while RAS causing reduced susceptibility to at least one DAA are marked with open symbols. Identified transmission pairs are highlighted grey, while transmission pairs identified consistently across all genomic regions are highlighted yellow.
